# Editorial: Role of the Platelet Phenomenon in Cardiovascular Diseases and Cancer

**DOI:** 10.3389/fcvm.2022.859770

**Published:** 2022-03-23

**Authors:** Marina Panova-Noeva, Anna Falanga

**Affiliations:** ^1^Center for Thrombosis and Hemostasis (CTH), University Medical Center of the Johannes Gutenberg University Mainz, Mainz, Germany; ^2^Department of Medicine and Surgery, University of Milan Bicocca, Monza, Italy; ^3^Department of Transfusion Medicine and Hematology, Hospital Papa Giovanni XXIII, Bergamo, Italy

**Keywords:** venous thromboembolism, platelets, cardiovascular disease, arterial thromboembolism, cancer

For already a century venous thromboembolism (VTE) has been established as one of the leading causes of morbidity and mortality in cancer patients. Compared to the general population, cancer patients have between 12- and 23-fold higher risk for VTE, the later calculated for cancer patients receiving anticancer therapy (1). Cancer patients are complex in terms of VTE management considering their increased risk for both recurrent thrombosis and bleeding complications (2). Unlike VTE, arterial thromboembolism (ATE) has only recently began to receive an increased attention in cancer patients. In presence of cancer, the risk of an ATE is approximately 3-fold higher compared to cancer-free patients (3). Platelet activation has an undisputed role in atherothrombosis, after an atherosclerotic plaque rupture or erosion, typically leading to the most common cardiovascular events as myocardial infarction (MI) or ischemic stroke (4). After an incident arterial thrombosis, the dual antiplatelet regimen, combination of cyclooxygenase inhibitor and P2Y12 antagonists, is the recommended approach for secondary prevention of adverse cardiovascular events. The management of cancer patients with an arterial thrombosis is particularly challenging due to scarce evidence for the best treatment approach in this patient population.

The selected papers in this Research Topic discuss the unmet needs of cancer patients presenting with an ATE and the mechanism behind increased risk of bleeding in cancer patients receiving a tyrosine kinase inhibitor. The potential of platelet biomarkers in predicting adverse cardiovascular events and platelet role in development of arterial hypertension has been further addressed. The authors additionally identify areas of research with open questions and challenges in this field.

The increased risk of an ATE in cancer patients is recognized as particularly high in the first 6 months following a cancer diagnosis (5). The interplay of multiple factors such as cancer type and stage, initiation of the antineoplastic treatment, the individual baseline risk for cardiovascular disease have been all implicated to have a role for an increased risk of ATE in cancer patients (6). Inohara et al. discuss the pathophysiological mechanisms, the clinical presentation, the available evidence and the clinical challenges on management of MI in cancer patients, with a focus on invasive strategy. Endothelial dysfunction in cancer patients has been recognized to have a prominent role facilitating platelet activation and aggregation resulting with an acute arterial thrombosis. The lack of solid evidence for the optimal treatment strategy in cancer patients at high risk for ATE stresses the need of future randomized clinical trials and prospective studies that will address many of the open questions in this vulnerable population.

In addition to thrombosis, cancer patients are as well at increased risk for bleeding. Tullemans et al., report the mechanisms for increased risk of bleeding in renal cell cancer patients treated with pazopanib, an angiostatic tyrosine kinase inhibitor (TKI). By *in-vitro* experiments with isolated platelets from healthy volunteers pazopanib addition was associated with strong inhibition of collagen-induced platelet activation, aggregation and phosphatidylserine (PS) exposure. The suppression of GPVI-mediated PS exposure was further confirmed in renal cell cancer patients treated with pazopanib that in combination with reduced platelet count, frequently observed in these patients, may explain the mild bleeding phenotype described during pazopanib treatment. This finding is particularly relevant when assessing bleeding risk in this patient population, especially when TKIs are prescribed in combination with antiplatelet agents.

Several platelet biomarkers have been tested for association with cardiovascular events from a low-cost and easy to measure mean platelet volume (MPV) to more complex platelet function tests necessitating special equipment and skilled personnel. Patients with coronary artery disease (CAD) despite the intake of antiplatelet agents remain at increased risk for CVD (7). High on-treatment platelet reactivity has been associated with higher risk for adverse cardiovascular outcome in patients with CAD, however the routine measurements remain challenging and not widely recommended (8). Zhao et al. address the value of reticulated platelets, as young immature platelet population with large size, metabolically active and indicator of increased platelet turnover. The authors propose that circulating levels of reticulated platelets could be used as an additional biomarker for prediction of cardiovascular events in CAD patients. Still, larger prospective studies are required to confirm the prognostic value of reticulated platelets in this patient population.

In addition to the well described role of platelets in the atherothrombotic process, platelet activation has been associated with all of the traditional cardiovascular risk factors, including the most prevalent in a general adult population, the arterial hypertension. The disturbed hemodynamics in arterial hypertension has been implicated to cause increased platelet activation that further increases the risk of thrombosis in these patients (9). In addition, activated platelets secrete a wide range of proinflammatory mediators that contribute to vascular inflammation and vessel wall remodeling, a known feature of arterial hypertension (10). To unravel if there is a causal effect between platelet count and arterial hypertension, Chiu et al., have performed a bi-directional Mendelian randomization (MR) analysis in 15,996 healthy Taiwanese individuals aged between 30 and 70 years with 388,331 single nucleotide polymorphisms (SNPs) used as the instrumental variables. The results of this study showed a positive causal effect of platelet count on the risk of hypertension, and no causal effect of hypertension on platelet count. In light of these findings, future mechanistic studies are needed to clarify the related biological pathways and pathogenic mechanisms linking platelet activation and arterial hypertension.

Overall, the collection in this Research Topic highlights platelets as an important link in cancer patients for both arterial thrombosis and bleeding complications. Further investigations should focus on elucidating and validating novel platelet-related biomarkers with clinical implication and predictive value for CVD.

## Author Contributions

MP-N prepared the editorial. AF critically revised its content. Both authors meet the ICMJE criteria for authorship for this manuscript, take responsibility for the integrity of the work as a whole, and have given final approval to the version to be published.

## Conflict of Interest

The authors declare that the research was conducted in the absence of any commercial or financial relationships that could be construed as a potential conflict of interest.

## Publisher's Note

All claims expressed in this article are solely those of the authors and do not necessarily represent those of their affiliated organizations, or those of the publisher, the editors and the reviewers. Any product that may be evaluated in this article, or claim that may be made by its manufacturer, is not guaranteed or endorsed by the publisher.
